# Upregulation of AT_1_ Receptor Mediates a Pressor Effect Through ROS-SAPK/JNK Signaling in Glutamatergic Neurons of Rostral Ventrolateral Medulla in Rats With Stress-Induced Hypertension

**DOI:** 10.3389/fphys.2018.01860

**Published:** 2019-01-08

**Authors:** Liping Jiang, Xuan Zhou, Hongyu Yang, Ruijuan Guan, Yanlei Xin, Jijiang Wang, Linlin Shen, Danian Zhu, Shulan Ma, Jin Wang

**Affiliations:** ^1^Department of Physiology and Pathophysiology, School of Basic Medical Sciences, Fudan University, Shanghai, China; ^2^Training Center of Medical Experiments, School of Basic Medical Sciences, Fudan University, Shanghai, China

**Keywords:** angiotensin II type 1 receptor, stress-induced hypertension, glutamatergic neurons, rostral ventrolateral medulla, NAPDH oxidase-ROS-SAPK/JNK signaling

## Abstract

The present study examined whether angiotensin II (Ang II) mediates the pressor effect through nicotinamide adenine dinucleotide phosphate (NADPH) oxidase-derived reactive oxygen species (ROS)-mitogen-activated protein kinase (MAPK) signaling in the glutamatergic neurons of the rostral ventrolateral medulla (RVLM) in stress-induced hypertensive rats (SIHR). The SIHR model was established using electric foot-shocks combined with noises for 15 days. We observed that Ang II type 1 receptor (AT_1_R) and the glutamatergic neurons co-localized in the RVLM of SIHR. Furthermore, glutamate levels in the intermediolateral column of the spinal cord were higher in SIHR than in controls. Microinjection of Ang II into the RVLM of SIHR activated stress-activated protein kinase/Jun N-terminal kinase (SAPK/JNK), extracellular signal-regulated protein kinase (ERK) 1/2, and p38MAPK. Compared with controls, the activation of SAPK/JNK, ERK1/2, p38MAPK, and ROS in the RVLM were higher in SIHR, an effect that was blocked by an NADPH oxidase inhibitor (apocynin) and an AT_1_R antagonist (candesartan). RVLM microinjection of apocynin or a SAPK/JNK inhibitor (SP600125), but not an ERK1/2 inhibitor (U0126) or a p38MAPK inhibitor (SB203580), decreased AT_1_R mRNA and mean arterial blood pressure (MABP) in SIHR. The increase of AT_1_R protein expression and MABP was inhibited by intracerebroventricular infusion (ICV), for 14 days, of SP600125, but not U0126 or SB203580 in SIHR. We conclude that Ang II modulates the pressor effect through AT_1_R-dependent ROS-SAPK/JNK signaling in glutamatergic neurons in the RVLM of SIHR.

## Introduction

Hypertension is a silent killer worldwide and an outcome of a convoluted interaction between genetic and environmental factors. The psychosocial stress caused by the fast-paced lifestyle of modern society, is an important risk factor for hypertension. Chronic stress leads to the generation of stress-induced hypertension (SIH) (Xia et al., 2008; Xiao et al., 2013; Zhang et al., 2013). Angiotensin II (Ang II) has important effects on the central modulation of cardiovascular activities. Ang II modulates sympathetic functions acting on a diverse range of receptors such as Ang II type 1 receptor (AT_1_R) and type 2 receptor (AT_2_R); the pressor effect of Ang II mainly results from the activation of AT_1_R (Reja et al., 2006).

Oxidative stress is caused by the imbalance between free radicals and antioxidants—an essential factor in hypertension. It has been indicated that nicotinamide adenine dinucleotide phosphate (NADPH) oxidase-derived reactive oxygen species (ROS), particularly superoxide anions (O_2_^.-^), are crucial intracellular messengers within Ang II signaling pathways (Hanna et al., 2002; Ushio-Fukai and Alexander, 2004). Ang II can upregulate mRNA and protein expression of NADPH oxidase subunits and augment production of ROS (Hausding et al., 2013; Wakui et al., 2013; Ding et al., 2015; Minas et al., 2015). In addition, ROS have been reported to participate in the modulation of sympathetic activities (Wang et al., 2002; Gao et al., 2004; Campese et al., 2005). NADPH oxidase-derived ROS act as essential intracellular messengers to activate downstream signaling molecules, including mitogen-activated protein kinase (MAPK) and transcriptional factors (Zhang et al., 2007; Bae et al., 2011; Lassegue et al., 2012). It is reported that Ang II activates stress-activated protein kinase/Jun N-terminal kinase (SAPK/JNK), extracellular signal-regulated protein kinase 1/2 (ERK1/2), and p38MAPK, which are critical protein kinases for gene expression and cell growth (Omura et al., 2005). A previous study demonstrated that chronic Ang II infusion elevated NADPH oxidase subunit protein expression, ROS production, as well as p38MAPK activation, leading to hypertension in Sprague Dawley rats (Bao et al., 2007).

The rostral ventrolateral medulla (RVLM) has an important impact on the maintenance of sympathetic activities. It is widely accepted that the neurons in the RVLM provide downward impulses to the sympathetic preganglionic neurons in the intermediolateral column (IML) of the spinal cord, which projects the sympathetic nerves that dominate the heart and blood vessels (Xiang et al., 2014). Our previous studies suggested that Ang II might activate glutamatergic neurons in the RVLM, resulting in the enhancement of blood pressure (BP) in Wistar and spontaneously hypertensive rats (SHR) (Hu et al., 2002). Moreover, AT1R, an angiotensin converting enzyme, mRNA and protein expression were significantly higher in the RVLM in stress-induced hypertensive rats (SIHR) compared with control rats (Du et al., 2013). It has been shown that the elevated glutamatergic inputs to the RVLM contribute to increased BP and sympathetic activities in Goldblatt hypertension (Carvalho et al., 2003) and spontaneous hypertension (Zha et al., 2013). However, the cellular and molecular mechanisms underlying the central roles of Ang II in SIHR remain to be identified. Therefore, this study evaluated the hypothesis that Ang II mediates pressor response through AT1R-dependent NADPH oxidase derived-ROS-MAPK signaling pathways in the glutamatergic neurons of the RVLM of SIHR.

## Materials and Methods

### Animals

Adult male Sprague-Dawley rats (200–220 g) were randomly housed (5 per cage) at room temperature (22–27°C), on a 12/12 h light/dark schedule, and allowed free access to food and water. All experimental procedures were approved by the Animal Use and Care Committee of Shanghai Medical College, Fudan University and were performed in strict accordance with the guidelines of the National Institutes Health Guide for the Care and Use of Laboratory Animals.

### Stress-Induced Hypertension Model

Rats were randomly allocated into normotension (control) and stressed-induced hypertension (SIH) groups. The SIH model was created as described in our previous studies (Xia et al., 2008; Xiao et al., 2013; Zhang et al., 2013). Briefly, the rats of SIH group were placed in a cage with a grid floor and received 2-h random electric foot-shocks paired with interval noise stimulation, twice daily, for 15 days. The control rats received the same treatment excluding the stressful stimuli. Systolic blood pressure (SBP) in conscious rats was measured using the tail-cuff method at 2 h after stress and obtaining the average of three measurements. The criterion for hypertension in this experiment was determined as an SBP greater than 140 mmHg. After the 15-day stimulation period, the rats in the SIH group with an SBP less than 140 mmHg were removed from the follow-up experiments.

### MABP Measurements

After endotracheal intubation, the right carotid artery of each rat was cannulated to monitor BP continuously using a polygraph (Model SMUP-A, Department of Physiology and Pathophysiology, Shanghai Medical College of Fudan University, Shanghai, China). The heart rate (HR) was derived automatically from the BP phasic wave. During the experiment, the rectal temperature of rats was kept at 37 ± 0.5°C using a thermostat.

### RVLM Microinjections

Similar to the procedures reported previously (Xiao et al., 2013), the rats were anesthetized with urethane/α-chloralose (urethane 0.75 g/kg; α-chloralose 70 mg/kg) intraperitoneally, and the bilaterally microinjection of test agents to the RVLM was performed with a micropipette (40–70 μm internal diameter) attached to a 0.5 μl microsyringe (Hamilton). The RVLM was located 0.8 mm from the first anterior branch of the hypoglossal nerve, 1.9 mm lateral, and 0.7 mm deeper toward the ventral surface. As a routine, a total volume of 50 nl was delivered to each side of the RVLM over 30 s to allow for complete diffusion of the test agents. The RVLM injection was verified by the presence of a transient pressor response (MABP greater than 15 mmHg) to an L-glutamate (2 nmol/50 nl) microinjection (Ross et al., 1984). Ang II, AT_1_R antagonist candesartan, and NADPH oxidase inhibitor apocynin, were purchased from Sigma-Aldrich (St. Louis, MO, United States). The SAPK inhibitor SP600125, ERK1/2 inhibitor U0126, and p38MAPK inhibitor SB203580 were obtained from Sigma-Aldrich (St. Louis, MO, United States). Artificial cerebrospinal fluid (aCSF) or 1% dimethyl sulphoxide (the solvent of SP600125, U0126, or SB203580) was used as a vehicle control. To avoid the confounding effects of drug interactions, each animal received only one pharmacological treatment. After the experiments, 50 nl of pontamine sky blue was injected through the micropipette to confirm the position (rostroventrolateral reticular nucleus, lateral paragigantocellular nucleus).

### Fluorescence *in situ* Hybridization (FISH)

The staining procedure was performed with an enhanced sensitive FISH detection kit (Servicebio, China). Six-micron cryosections were prepared ahead of time. We applied proteinase K (20 μg/ml) on the slice for 5 min to expose the nucleic acid and then washed the slides three times with 0.5M PBS. Following incubation with hybridization buffer at 37°C for 2 h, the slides were incubated with 50 μl of 8 ng/ul 5′,3′ double CY3-labeled RNA (VGLUT2 mRNA) detection probe and 5′,3′ double FAM-labeled RNA (AT_1_R mRNA) detection probe (TSINGKE, China) in the same buffer at 37°C overnight. After the samples were washed with different concentrations of SSC buffers. After three 5 min washes in PBS for the last time, the slides were detected under a Nikon Ni-U microscope (Nikon, Japan) and pictures were collected.

### Intracerebroventricular (ICV) Infusion

Anesthetized rats were fixed in a stereotaxic apparatus (NeruoStar, United States). After the bregma was identified, an ICV cannula was implanted for the infusion of the inhibitors, as described previously (Dange et al., 2014). Once the anterior fontanel was identified, an ICV cannula was implanted into the right lateral cerebral ventricle 0.8 mm caudal to bregma, 1.5 mm lateral to the bregma, and 3.8 mm ventral to the zero level (Pan et al., 2007). The cannula was fixed to the cranium using dental acrylic and two stainless steel screws. The pump body was implanted subcutaneously. The rats underwent a 14-day ICV infusion of the SAPK/JNK inhibitor (SP600125), ERK1/2 inhibitor (U0126), p38MAPK inhibitor (SB203580) (0.5 μl/hr, 0.6 mmol/L), NADPH oxidase inhibitor APO (0.5 μl/hr, 0.45 mmol/L) (Fang et al., 2013), or aCSF via osmotic micro-pumps (Alzet, Cupertino, CA, United States). The position of the cannula in the lateral cerebral ventricle was confirmed by the staining of all four ventricles after injection of 5 μl of evans blue dye at the end of the experiments.

### Intermediolateral Column (IML) Microdialysis

After making an incision in the back to expose the T8 level of the thoracic segment and then removing the bone and meningeal membrane to expose the spinal cord surface, the microdialysis probe was inserted into the T8 spinal cord 0.45 mm lateral to the midline and 0.9 mm below the dorsal surface. The tip of the probe was in the IML. The probe was perfused (2 μl/min) with aCSF using a microdialysis pump (BASi, United States). Each dialysate sample was harvested for 10 min at a volume of 20 μl.

### High-Performance Liquid Chromatography (HPLC)

Amino acids of dialysate samples were separated using reverse-phase HPLC (Waters 1525, United States) and fluorescence detection (Waters 2475, United States) with a reverse-phase column (C18, ultrasphere octadecyl silane). The derivatized reagent consisted of 5 ml of absolute ethanol, 5 ml of 0.1 mol/L sodium tetraborate, 27 mg of orthophthaldialdehyde (Sigma-Aldrich, St. Louis, MO, United States), and 40 μl of β-mercaptoethanol (Sigma-Aldrich, St. Louis, MO, United States). The sample (20 μl) was mixed with the derivatized reagent (10 μl) for 90 s and then injected into the HPLC system. The mobile phase, which contained 63% 0.1 mol/L KH_2_PO4, 35% methanol, and 2% tetrahydrofuran, flowed through the system with a flow rate of 1.0 ml/min. The temperature of the column was 35°C. The excitation and emission wave lengths were 330 and 450 nm, respectively. For each sample, the analysis time was not more than 10 min.

### Collection of Tissue Samples From the RVLM

On the 15th day, or after microinjections, rats were euthanized and their brains were removed and frozen on dry ice. Both sides of the ventrolateral medulla covering the RVLM (0.5–2.5 mm rostral to the obex and 1.4–2.4 mm lateral to the midline and medial to the spinal trigeminal tract) were dissected using a stainless steel micropunch (1 mm internal diameter) (Chan et al., 2007). Medullary tissues were stored at –80°C prior to protein analysis.

### Western Blot Analysis

The samples of brain tissues were homogenized in RIPA lysis buffer with protease or phosphatase inhibitor (Roche, Basel, Switzerland) and then centrifuged (12000 rpm, 15 min, 4°C) to obtain supernatants. The total protein concentration of the supernatants was determined using the BCA assay kit (Pierce). Samples were loaded and separated on the SDS–PAGE gel (40 μg per well). The protein was transferred to the PVDF membrane (Millipore, MA, United States), which was incubated overnight with primary antibodies at 4°C and then probed with the corresponding secondary antibody. The primary antibodies were as follows: rabbit anti-AT_1_R antibody (1:800 dilution, Abcam, MA, United States) (Czikora et al., 2015; Wang et al., 2018), rabbit anti-SAPK/JNK antibody, mouse anti-phospho-SAPK/JNK antibody, mouse anti-ERK1/2 antibody, rabbit anti-phospho-ERK1/2 antibody, rabbit anti-p38MAPK antibody, rabbit anti-phospho-p38MAPK antibody and rabbit anti-β-tublin antibody (1:1000 dilution, Cell Signaling Technology, Danvers, MA, United States). The membranes were detected by an ECL-Plus detection kit (Tiangen, Beijing, China), and scanned using Image Quant LAS 4000 (GE Healthcare Life Sciences, CT, United States). The images were quantified using the ImageJ densitometry system.

### Measurement of ROS in the RVLM

The lucigenin-enhanced chemiluminescence assay was adopted to detect the ROS production in the RVLM (Chan et al., 2005). RVLM was homogenized in PBS (20 mM, pH 7.4) containing EDTA (0.01 mM). The homogenate was centrifuged at 1200 × *g* for 10 min at 4°C and the supernatant was collected for ROS assay. Background chemiluminescence in a 2-ml buffer mixed with lucigenin (5 μmol/L) was detected for 5 min. Adding 100 μl supernatant, the chemiluminescence was detected for 30 min at room temperature. The production of ROS was determined and expressed as average light units per minute per milligram of protein.

### Immunohistochemistry

The procedure for immunohistochemistry was described previously (Peng et al., 2009). One section from every six serial sections was picked up, with a total of five sections in each animal. Tissue sections were deparaffinized, rehydrated by graded ethanol series, pre-incubated with 5% BSA for an hour, and then incubated with mouse anti-phospho-SAPK/JNK antibody (1:50 dilution, Cell Signaling Technology, Danvers, MA, United States), rabbit anti-phospho-ERK1/2 antibody (1:200 dilution, Cell Signaling Technology, Danvers, MA, United States), or rabbit anti-phospho-p38MAPK antibody (1:400 dilution, Cell Signaling Technology, Danvers, MA, United States) at 4°C overnight. The sections were washed three times with PBS and then incubated with corresponding secondary antibodies. The color reaction was carried out with HRP-linked polymer detection system.

### Quantitative Real-Time PCR

The total RNA was extracted from lung tissues using TRIzol Reagent (Invitrogen Corporation, CA, United States). First-strand cDNA was synthesized and amplified from 0.5 μg of total RNA using the ReverTra Ace qPCR RT Kit (Toyobo, Tokyo, Japan). Then the mRNA levels of AT_1_R were measured by quantitative real-time PCR (iCyler iQ Real-time PCR Detection System, Bio-Rad Laboratories Inc., United States) using SYBR Green Real-time PCR Master Mix (Toyobo, Japan) in a total volume of 20 μL. Glyceraldehyde-3-phosphate dehydrogenase (GAPDH) was used as an internal standard to normalize the expression level of each mRNA. Primers were designed by Sangon Biotech (Shanghai, China). The target gene names and their primer sequences were shown as follows: forward primer 5′-CCCAAGTCCACACATCAAAG-3′, reverse primer 5′-GCAAGGCAGACTGTATGGAA-3′ for AT_1_R; and forward primer 5′-AAGGTGGTGAAGCAGGCGGC-3′, reverse primer 5′-GAGCAATGCCAGCCCCAGCA-3′ for GAPDH. The PCR amplification consisted of 40 cycles of denaturation (94°C, 15 s), annealing (60°C, 30 s) and extension (72°C, 30 s). All the samples were assayed in one essay in our study. The relative quantification of gene expression was analyzed from the measured threshold cycles (CT) by using the 2-ΔΔCt method in the experiment.

### Statistical Analysis

All of the data are presented as mean ± standard error (SE). Student’s *t*-test, paired *t*-test, or analysis of variance (ANOVA) was performed as appropriate. Statistical analyses were performed using Statistical Package for the Social Sciences (SPSS) version 16.0. A *P* < 0.05 was considered statistically significant.

## Results

### The Systolic Blood Pressure (SBP) and Heart Rate (HR) of the Stressed Rats Increase in a Time-Dependent Manner

**FIGURE 1:**
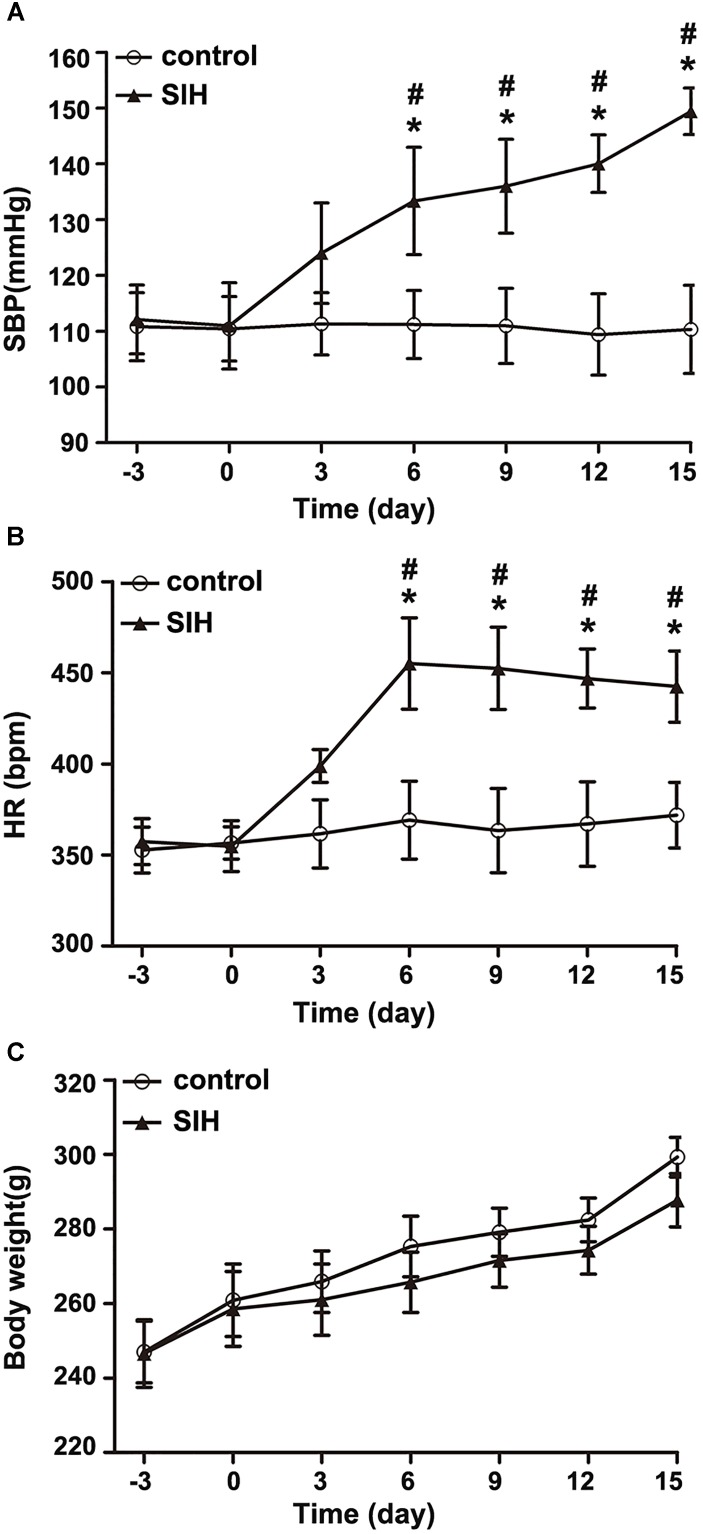
The changes of systolic blood pressure (SBP) **(A)**, heart rate (HR) **(B)**, and body weight **(C)** in stress-induced hypertension (SIH) and control group. *n* = 10, *^∗^P* < 0.05 vs. control (Student’s *t*-test); ^#^*P* < 0.05 vs. baseline (paired *t*-test) (third day prior to stress).

Systolic blood pressure and HR of the stressed rats significantly increased from the 6th day compared with the control rats (^∗^*P* < 0.05). SBP and HR of stressed rats was also significantly different from the 6th day compared with baseline (3rd day prior to stress) (^#^*P* < 0.05), and stayed stable at a high level around the 15th day (Figures 1A,B). However, there was no discernible difference in body weight between the SIH and control group (Figure 1C).

### AT_1_R and VGLUT2 Co-localize in the RVLM and the Release of Glutamate Increases in the IML of SIHR

**FIGURE 2:**
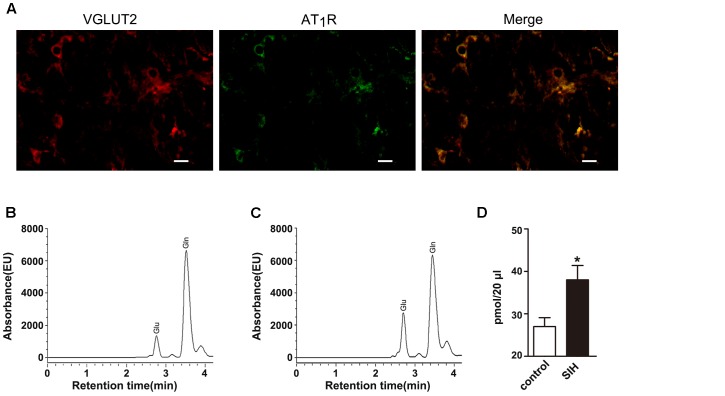
Localization of AT_1_R and VGLUT2 in the rostral ventrolateral medulla (RVLM) of SIH rat **(A)** and chromatogram of amino acids in the IML in control **(B)** and SIH rats **(C)** the red (cy3), green (FAM) areas represented the staining of glutamatergic neurons and AT_1_R, respectively. Scale bars = 25 μm. The baseline release of glutamate increased in SIH rats compared with control rats **(D)**. *n* = 8, ^∗^*P* < 0.05 vs. control (Student’s *t*-test). Glu, glutamate; Gln, glutamine.

Figure 2A showed that AT_1_R and VGLUT2 co-localized in the RVLM of SIHR, suggesting that AT_1_R expressed in the glutamatergic neurons. To establish that the development of SIH is associated with the actions of glutamatergic neurons in the RVLM, we examined the concentration of amino acid neurotransmitters in the IML. We found the release of glutamate (Glu) neurotransmitter increased in SIHR (Figure 2C) than in the control rat (Figure 2B) (*n* = 8, *P* < 0.05, Figure 2D). These data reveal that the activated glutamatergic neurons of the RVLM release more glutamate neurotransmitter to the IML of SIHR.

### Exogenous Ang II Activates SAPK/JNK, ERK1/2 and p38MAPK in the RVLM of SIHR

**FIGURE 3:**
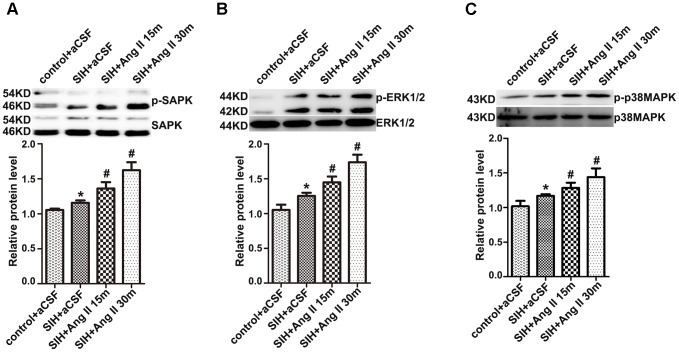
The protein levels of phosphorylation or total SAPK/JNK **(A)**, ERK1/2 **(B)**, and p38MAPK **(C)** in the RVLM in the SIH group 15 and 30 min after RVLM microinjection of Ang II (50 pmol). Densitometry values were determined as indicated. *n* = 4, ^∗^*P* < 0.05 vs. control; ^#^*P* < 0.05 vs. SIH (one-way ANOVA with Tukey *post hoc* test).

As Figure 3 shows, SAPK/JNK, ERK1/2, and p38MAPK phosphorylation in the RVLM were much higher in SIHR than in control rats. RVLM microinjection of Ang II (50 pmol) increased SAPK/JNK, ERK1/2, and p38MAPK phosphorylation in SIHR at 15 min after application, and lasted at least 30 min; while total SAPK/JNK, ERK1/2, or p38MAPK levels were unchanged (Figures 3A–C).

### Antagonist of AT_1_R Decreases ROS Production and SAPK/JNK, ERK1/2, and p38MAPK Phosphorylation in the RVLM of SIHR

**FIGURE 4:**
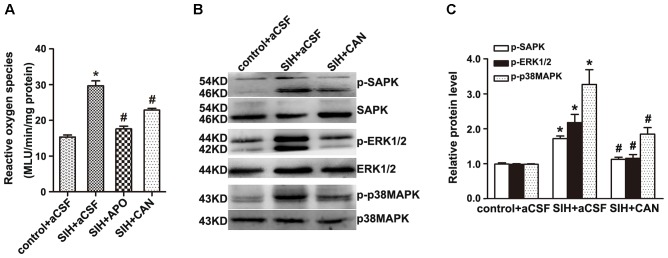
Effects of candesartan (CAN) on the production of Reactive oxygen species (ROS) and phosphorylation of SAPK/JNK, ERK1/2, and p38MAPK in the RVLM in SIH and control groups. The production of ROS in the RVLM detected at 20 min after microinjection of apocynin APO (2 nmol) or CAN (2 nmol) into the RVLM **(A)**; *n* = 6. The protein levels of phosphorylated SAPK/JNK, ERK1/2, and p38MAPK were detected 30 min after microinjection of CAN (2 nmol) into the RVLM, bilaterally **(B,C)**; *n* = 4. ^∗^*P* < 0.05 vs. control + aCSF; ^#^*P* < 0.05 vs. SIH + aCSF (one-way ANOVA with Tukey *post hoc* test).

The production of ROS was significantly higher in the RVLM of SIHR (Figure 4A). Furthermore, the enhancement of ROS production and SAPK/JNK, ERK1/2, and p38MAPK phosphorylation were discernibly blunted (Figures 4B,C) by administration of candesartan (2 nmol, an AT_1_R antagonist) to the RVLM, bilaterally. However, there was no discernible change elicited by microinjection of PD123319 (2 nmol, an AT_2_R antagonist) to the RVLM, bilaterally (data not shown). These findings implicate that stress elevates phosphorylation of SAPK/JNK, ERK1/2, and p38MAPK and activates MAPK pathway via AT_1_R.

### NADPH Oxidase Inhibitor Inhibits the Activation of MAPK in the RVLM of SIHR

**FIGURE 5:**
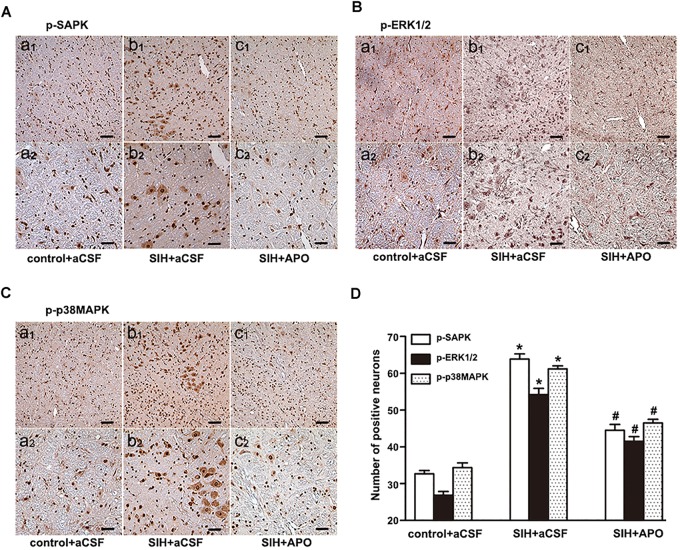
Effects of APO on MAPK activation in the RVLM in the SIH and control group. Immunohistochemistry for p-SAPK/JNK **(A)**, p-ERK1/2 **(B)**, or p-p38MAPK **(C)** in control and SIH groups treated with or without APO **(D)**. Scale bars = 50 μm in a1, b1, c1; 25 μm in a2, b2, c2. *n* = 4, ^∗^*P* < 0.05 vs. control + aCSF; ^#^*P* < 0.05 vs. SIH + aCSF (one-way ANOVA with Tukey *post hoc* test).

Bilateral administration of NADPH oxidase inhibitor APO (2 nmol) significantly inhibits SAPK/JNK, ERK1/2, or p38MAPK activation in the RVLM of SIHR (Figures 5A–D). These findings suggest that NADPH oxidase-derived ROS might be an upstream signaling molecule within the MAPK pathway.

### Inhibitor of SAPK, but Not ERK1/2 or p38MAPK, Inhibits AT_1_R Expression in the RVLM of SIHR

**FIGURE 6:**
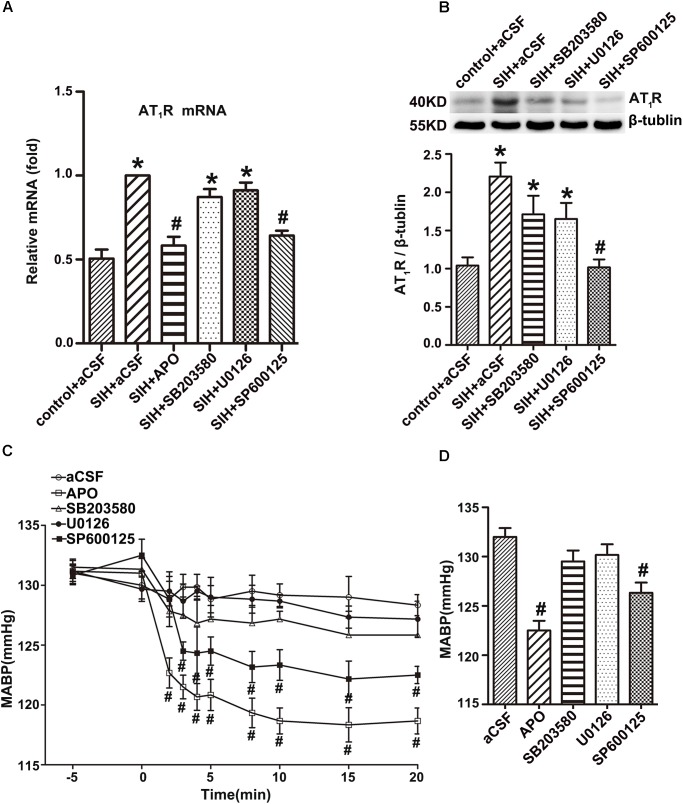
Effects of the inhibitors of nicotinamide adenine dinucleotide phosphate (NADPH) oxidase, SAPK/JNK, ERK1/2, and p38MAPK on AT_1_R expression in the RVLM and Mean arterial blood pressure (MABP) in rats with SIH. The mRNA levels of AT_1_R in the RVLM (*n* = 4) **(A)**, and change in MABP (*n* = 6) **(C)** in response to microinjection of APO (2 nmol), SP600125 (500 nmol), U0126 (500 nmol), or SB203580 (500 nmol) into the RVLM of rats with SIH. Representative gels or densitometric analysis of AT_1_R protein in the RVLM (*n* = 5) **(B)**, and change in MABP (*n* = 6) **(D)** of SIH group after intracerebroventricular infusion (ICV) of aCSF, APO (0.45 mol/L), SP600125 (0.6 mol/L), U0126 (0.6 mol/L), or SB203580 (0.6 mol/L) for 14 days in stress-induced hypertensive rats (SIHR). ^∗^*P* < 0.05 vs. control + aCSF; ^#^*P* < 0.05 vs. SIH + aCSF (one-way ANOVA with Tukey *post hoc* test, **A,B,D**; Student’s *t*-test, **C**).

Both RT-PCR and Western blot studies showed that APO or SAPK inhibitor SP600125 led to a profound decrease in AT_1_R mRNA and protein expression in the RVLM of SIHR, whereas ERK1/2 inhibitor U0126 or p38MAPK inhibitor SB203580 did not demonstrate a significant effect in AT_1_R mRNA and protein expression in the RVLM of SIHR (Figures 6A,B).

### Inhibitors of NADPH Oxidase and SAPK Decreased MABP of SIHR

Bilateral microinjection of the APO (2 nmol) or the SAPK inhibitor SP600125 (500 nmol) into the RVLM decreased MABP of SIHR. However, administration of the ERK1/2 inhibitor U0126 (500 nmol) or p38MAPK inhibitor SB203580 (500 nmol) into the RVLM did not obviously change the MABP of SIHR (Figure 6C). There was no obvious change in the HR of SIHR. In addition, microinjection of SP600125, U0126, or SB203580 into regions adjacent to the RVLM had no discernible change in the MABP or HR of SIHR. ICV infusion of the APO (0.45 mmol/L) or SP600125 (0.6 mmol/L) for 14 days decreased MABP in the SIHR, whereas U0126 (0.6 mmol/L) or SB203580 (0.6 mmol/L) did not obviously change the MABP of SIHR (Figure 6D).

## Discussion

Hypertension is a major risk factor for cardiovascular and cerebrovascular diseases, which affects over one billion individuals worldwide (Wu et al., 2015; Mills et al., 2016). SIH caused by chronic and excessive stress, is inflicted on an increasing number of people, including young adults. Therefore, it is necessary to understand the mechanism underlying SIH. The SIHR model is established by electric foot-shocks combined with interval noises (Xia et al., 2008; Xiao et al., 2013; Zhang et al., 2013). The present experiment revealed that this chronic stress in rats led to an increase in BP, implying the successful establishment of an SIHR model.

In the current study, we examined the roles of AT_1_ R on cardiovascular modulation and the effects of NADPH oxidase-derived ROS-MAPK signaling in the glutamatergic neurons of the RVLM in SIHR. The primary findings of the present study are: (1) AT_1_R expressed in the glutamatergic neurons of the RVLM of SIHR and the glutamate in the IML of the spinal cord is higher in SIHR than that in control rats. (2) Exogenous Ang II activates SAPK/JNK, ERK1/2, and p38MAPK in the RVLM of SIHR. AT_1_R antagonist and NADPH oxidase inhibitor reduce the enhanced production of ROS and the activation of SAPK/JNK, ERK1/2, and p38MAPK in the RVLM of SIHR. (3) Injection of the SAPK/JNK inhibitor, but not the p38MAPK or the ERK1/2 inhibitor, into the RVLM significantly decreases expression of AT_1_R mRNA and MABP in SIHR. ICV infusion of the SAPK/JNK inhibitor for 14 days, but not the p38MAPK or the ERK1/2 inhibitor, significantly decreases the expression of AT_1_R protein and MABP in SIHR.

First, we identified that AT_1_R in glutamatergic neurons played a crucial role in the pathogenesis of SIH. Fluorescence *in situ* hybridization (FISH) analysis showed that the AT_1_R and VGLUT2 co-localized in the RVLM of SIHR, which indicated that the activity that Ang II bands to AT_1_R in the RVLM, is connected to the glutamatergic neurons. A majority of the glutamatergic neurons were co-localized with AT_1_R in the RVLM of Wistar rats and SHR (Hu et al., 2002). In hypertensive Dahl salt-sensitive rats, the elevated BP response to AT_1_R activation in the hypothalamic paraventricular nucleus (PVN) has been shown to be partly regulated by increased glutamate receptor activation (Gabor and Leenen, 2012). Ang II infusion increases co-localization of gp91^phox^-containing NADPH oxidase and glutamate N-methyl-D-aspartate receptor (NMDAR) NR_1_ subunit in the dendrites of PVN neurons, and augments baseline and NMDAR-induced production of ROS in PVN cells and spinally projecting PVN neurons (Wang et al., 2013). Our findings provide evidence that AT_1_R expressed in the glutamatergic neurons of the RVLM in SIHR, indicating that Ang II might evoke the activation of glutamatergic neurons via AT_1_R in the RVLM of SIHR. Furthermore, HPLC analysis showed that the glutamate was increased in the IML of spinal cord. It has been shown that projections from the RVLM to the IML of spinal cord dominate preganglionic neurons resulting in the elevated BP and HR (Guyenet, 2006; Oshima et al., 2008). Our previous studies determined that Ang II-induced glutamate release in the spinal cord might arise from the AT_1_R-containing glutamatergic spinally projecting neurons in the RVLM of SHR (Hu et al., 2002). Our present findings implicate that, in SIHR, the glutamatergic neurons of the RVLM are activated, which are associated with the binding of Ang II to AT_1_R, and the further release of more glutamate neurotransmitter into the IML through projection fibers. The vesicular glutamate transporters (VGLUTs), capable of specifically packaging glutamate into presynaptic vesicles and promoting glutamate release (Liguz-Lecznar and Skangiel-Kramska, 2007), comprise three members, wherein VGLUT1 and VGLUT2 are considered as specific markers of canonical glutamatergic neurons. Additionally, VGLUT2-immunoreactivities were widely observed in the lower brainstem (Vigneault et al., 2015).

Chronic infusion of Ang II into the brain of normal rabbits resulted in sympathoexcitation and increased oxidative stress, as well as an upregulation of several of the protein subunits of NADPH oxidase, which have shown to be inhibited by losartan (Gao et al., 2005). In a study performed in rabbits with chronic heart failure, central administration of losartan, superoxide dismutase mimetic tempol, or NADPH oxidase inhibitor APO, reduced sympathetic nerve activity and oxidative stress (Gao et al., 2004). What is more, other models demonstrated similar findings, such as cardiac diastolic dysfunction (Li et al., 2013) and nitric oxide inhibition induced-hypertension (Rincón et al., 2015). In our experiments, ICV infusion for 14 days or RVLM microinjection of NADPH oxidase inhibitor APO obviously decreased MABP in the SIHR. These results implicate that AT_1_R and oxidative stress elicited by NADPH oxidase have an important effect on the regulation of sympathetic activities.

Secondly, we observed that the MAPK pathway was activated in SIHR and, specifically, that Ang II activated the MAPK pathway via AT_1_R. The current data show that SAPK/JNK, ERK1/2, and p38MAPK phosphorylation in the RVLM were significantly higher in SIHR than in control rats. Additionally, exogenous Ang II application to the RVLM causes this increase in SAPK/JNK, ERK1/2, and p38MAPK phosphorylation in SIHR. The enhanced activation of MAPKs has been observed in pulmonary arterial hypertension and spontaneous hypertension (Cao et al., 2014; Awad et al., 2016). Importantly, we found that the activation of SAPK/JNK, ERK1/2, and p38MAPK in the RVLM was significantly inhibited by bilateral microinjection of AT_1_R antagonist candesartan, which can cross the blood-brain barrier easily.

Our findings might not be consistent with previous studies noting that in normal rats, Ang II elicited ERK1/2 and p38MAPK phosphorylation, whereas SAPK/JNK was not activated in normal rats (Chan et al., 2005). The difference between the current study and these previous results is that SAPK/JNK, ERK1/2, and p38MAPK phosphorylation induced by Ang II administration may be related to the specialty of the current SIH model. Chronic and excessive stress can activate not only ERK1/2 and p38MAPK, but also the stress related proteins SAPK/JNK, to regulate sympathetic activity further.

Thirdly, we demonstrated that NADPH oxidase-derived ROS is the upstream messenger that mediated SAPK/JNK, ERK1/2, and p38MAPK activation induced by Ang II binding to AT_1_R in the RVLM of SIHR. We identified that the production of ROS enhanced in SIHR compared with control rats. Microinjections of AT_1_R antagonist candesartan and NADPH oxidase inhibitor APO into the RVLM partially inhibited ROS production and SAPK/JNK, ERK1/2, and p38MAPK phosphorylation in SIHR. APO also inhibited the elevation of MABP in SIHR. A previous study suggested that NADPH oxidase-derived ROS mediated the Ang II-evoked pressor effect via p38MAPK activation in the RVLM of normal rats (Chan et al., 2005). The present study suggests that in the RVLM of SIHR, NADPH oxidase-derived ROS has a crucial effect on SAPK/JNK, ERK1/2, and p38MAPK activation elicited by endogenous Ang II.

Finally, we noted that SAPK/JNK activation in the RVLM had important effects on AT_1_R expression and the modulation of pressor response. The enhancement of AT_1_R mRNA expression and MABP in SIHR were attenuated by the bilateral microinjection of SAPK/JNK inhibitor SP600125, but not ERK1/2 inhibitor U0126, or p38MAPK inhibitor SB203580. Besides, ICV infusion of the SAPK/JNK inhibitor, not ERK1/2, or p38MAPK inhibitor for 14 days decreased AT_1_R protein expression and MABP in the SIHR, Our previous study demonstrated that endogenous Ang II production was increased in SIHR and AT_1_R expression in the RVLM was much higher in SIHR than in control rats (Du et al., 2013). Hence, we suggested that SAPK/JNK played a major role in regulating the pressor effect.

**FIGURE 7:**
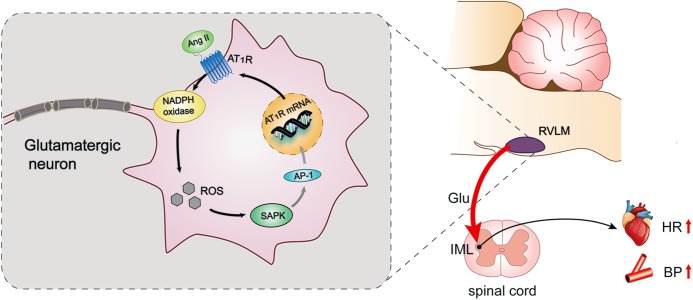
Schematic diagram showing the mechanism by which AT_1_R mediates pressor effect in the glutamatergic neurons of the RVLM of SIHR. Ang II triggers the activation of NADPH oxidase derived ROS-SAPK pathway via AT_1_R in the glutamatergic neurons of the RVLM of SIHR, then may activate activator protein (AP)-1 to evoke gene transcription and increase AT_1_R expression, which promotes the release of glutamate in the IML and eventually increases BP and HR.

The AT_1_R expression was enhanced in the RVLM of normal rabbits infused with Ang II and rabbits with heart failure (Gao et al., 2005; Liu et al., 2006). It has been reported that Ang II induces MAPK signaling to upregulate AT_1_R in PVN and contributes to AT_1_R-mediated sympathetic excitation in heart failure (Wei et al., 2008). Ang II activates diverse nuclear transcription factors, such as activator protein-1 (AP-1) (Jia et al., 2008), some of which participate in AT_1_R gene transcription. There was some evidence that Ang II promoted the upregulated transcription of AT_1_R by oxidant stress and AP-1 activation in the intact brain of rabbits and cultured neuronal cells (Liu et al., 2008). The enhancement of AP-1 upregulated AT_1_R expression in the RVLM of rabbits with heart failure, which was activated by the SAPK/JNK pathway (Liu et al., 2006). Furthermore, Ang II-evoked ERK1/2 phosphorylation also functioned in central sympathetic activities via transcription regulation. For example, the PKCb/NADPH oxidase/ERK1/2/CREB/c-fos signaling cascade modulates the long-term pressor effect induced by Ang II in the RVLM of normal rats (Chan et al., 2007). Our present findings indicate that SAPK/JNK, but not ERK1/2 or p38MAPK, has a crucial effect on the modulation of pressor response and AT_1_R expression in the RVLM of SIHR. The findings reveal that Ang II upregulates its own receptors via positive feedback involving the AT_1_R-ROS- SAPK/JNK pathway (Figure 7).

Overall, the present data show that AT_1_R localizes in the glutamatergic neurons of the RVLM of SIHR. This localization results in the release of glutamate into the IML of the spinal cord, leading to the pressor response in SIHR. Further, the NADPH oxidase-ROS-SAPK/JNK pathway has an essential impact on the actions of glutamatergic neurons and promotes the expression of AT_1_R in the RVLM of SIHR. Thus, this study reveals that Ang II modulates pressor effect through AT_1_R-dependent activation of SAPK/JNK by NADPH oxidase-derived ROS in the glutamatergic neurons of the RVLM in SIHR. One limitation of the present study is that we didn’t examine the expression of AP-1 and the role of AP-1 in SIHR, although it has been reported that AP-1 activation might participate in the upregulation of AT_1_R in heart failure. This interesting possibility in SIHR will be investigated in our future study.

## Author Contributions

JinW designed the work. LJ, XZ, RG, and YX performed the experiments. XZ, JinW, and SM wrote the manuscript. HY drew the figures. JinW, JijW, LS, and DZ interpreted the data.

## Conflict of Interest Statement

The authors declare that the research was conducted in the absence of any commercial or financial relationships that could be construed as a potential conflict of interest.

## Abbreviations

aCSF: artificial cerebrospinal fluid
Ang II: angiotensin II
AP-1: activator protein-1
APO: apocynin
AT_1_R: angiotensin II type 1 receptors
BP: blood pressure
CAN: candesartan
FISH: fluorescence *in situ* hybridization
Glu: glutamate
HR: heart rate
ICV: intracerebroventricular
IML: intermediolateral column
MABP: mean arterial blood pressure
ROS: reactive oxygen species
RVLM: rostral ventrolateral medulla
SBP: systolic blood pressure
SHR: spontaneously hypertensive rats
SIH: stress-induced hypertension
SIHR: stress-induced hypertensive rats

## References

[B1] Awad K. S. West J. D. de Jesus P. V. MacLean M. (2016). Novel signaling pathways in pulmonary arterial hypertension (2015 Grover Conference Series). *Pulm. Circ.* 6 285–294. 10.1086/688034 27683605 PMC5019081

[B2] Bae Y. S. Oh H. Rhee S. G. Yoo Y. D. (2011). Regulation of reactive oxygen species generation in cell signaling. *Mol. Cells* 32 491–509. 10.1007/s10059-011-0276-3 22207195 PMC3887685

[B3] Bao W. Behm D. J. Nerurkar S. S. Ao Z. Bentley R. Mirabile R. C. (2007). Effects of p38 MAPK inhibitor on angiotensin II-dependent hypertension, organ damage, and superoxide anion production. *J. Cardiovasc. Pharmacol.* 49 362–368. 10.1097/fjc.0b013e318046f34a 17577100

[B4] Campese V. M. Shaohua Y. Huiquin Z. (2005). Oxidative stress mediates angiotensin II-dependent stimulation of sympathetic nerve activity. *Hypertension* 46 533–539. 10.1161/01.hyp.0000179088.57586.26 16116043

[B5] Cao Y. Zhang Y. Wang N. He L. (2014). Antioxidant effect of imperatorin from angelica dahurica in hypertension via inhibiting NADPH oxidase activation and MAPK pathway. *J. Am. Soc. Hypertens.* 8 527–536. 10.1016/j.jash.2014.04.006 24973900

[B6] Carvalho T. H. F. Bergamaschi C. T. Lopes O. U. Campos R. R. (2003). Role of endogenous angiotensin II on glutamatergic actions in the rostral ventrolateral medulla in goldblatt hypertensive rats. *Hypertension* 42 707–712. 10.1161/01.hyp.0000086524.35251.2D 12913058

[B7] Chan S. H. Hsu K. S. Huang C. C. Wang L. L. Ou C. C. Chan J. Y. (2005). NADPH oxidase-derived superoxide anion mediates angiotensin II-induced pressor effect via activation of p38 mitogen-activated protein kinase in the rostral ventrolateral medulla. *Circ. Res.* 97 772–780. 10.1161/01.res.0000185804.79157.C0 16151022

[B8] Chan S. H. Wang L. L. Tseng H. L. Chan J. Y. (2007). Upregulation of AT1 receptor gene on activation of protein kinase C beta/nicotinamide adenine dinucleotide diphosphate oxidase/ERK1/2/c-fos signaling cascade mediates long-term pressor effect of angiotensin II in rostral ventrolateral medulla. *J. Hypertens.* 25 1845–1861. 10.1097/hjh.0b013e328217b286 17762649

[B9] Czikora I. Feher A. Lucas R. Fulton D. J. Bagi Z. (2015). Caveolin-1 prevents sustained angiotensin II-induced resistance artery constriction and obesity-induced high blood pressure. *Am. J. Physiol. Heart Circ. Physiol.* 308 H376–H385. 10.1152/ajpheart.00649.2014 25527780 PMC4346763

[B10] Dange R. B. Agarwal D. Masson G. S. Vila J. Wilson B. Nair A. (2014). Central blockade of TLR4 improves cardiac function and attenuates myocardial inflammation in angiotensin II-induced hypertension. *Cardiovasc. Res.* 103 17–27. 10.1093/cvr/cvu067 24667851

[B11] Ding K. Wang Y. Jiang W. Zhang Y. Yin H. Fang Z. (2015). Qian yang yu yin granule-containing serum inhibits angiotensin II-induced proliferation, reactive oxygen species production, and inflammation in human mesangial cells via an NADPH oxidase 4-dependent pathway. *BMC Complement. Altern. Med.* 15:81. 10.1186/s12906-015-0619-2 25886843 PMC4387585

[B12] Du D. Chen J. Liu M. Zhu M. Jing H. Fang J. (2013). The effects of angiotensin II and angiotensin-(1-7) in the rostral ventrolateral medulla of rats on stress-induced hypertension. *PLoS One* 8:e70976. 10.1371/journal.pone.0070976 23967142 PMC3743893

[B13] Fang X. Shu G. Yu J. Wang L. Yang J. Zeng Q. (2013). The anorexigenic effect of serotonin is mediated by the generation of NADPH oxidase-dependent ROS. *PLoS One* 8:e53142. 10.1371/journal.pone.0053142 23326391 PMC3541393

[B14] Gabor A. Leenen F. H. H. (2012). Cardiovascular effects of angiotensin II and glutamate in the PVN of dahl salt-sensitive rats. *Brain Res.* 1447 28–37. 10.1016/j.brainres.2012.01.060 22356885

[B15] Gao L. Wang W. Li Y. L. Schultz H. D. Liu D. Cornish K. G. (2004). Superoxide mediates sympathoexcitation in heart failure: roles of angiotensin II and NAD(P)H oxidase. *Circ. Res.* 95 937–944. 10.1161/01.res.0000146676.04359.64 15459075

[B16] Gao L. Wang W. Li Y. L. Schultz H. D. Liu D. Cornish K. G. (2005). Sympathoexcitation by central ang II: roles for AT1 receptor upregulation and NAD(P)H oxidase in RVLM. *Am. J. Physiol. Heart Circ. Physiol.* 288 H2271–H2279. 10.1152/ajpheart.00949.2004 15637113

[B17] Guyenet P. G. (2006). The sympathetic control of blood pressure. *Nat. Rev. Neurosci.* 7 335–346. 10.1038/nrn1902 16760914

[B18] Hanna I. R. Taniyama Y. Szocs K. Rocic P. Griendling K. K. (2002). NAD(P)H oxidase-derived reactive oxygen species as mediators of angiotensin II signaling. *Antioxid. Redox. Signal.* 4 899–914. 10.1089/152308602762197443 12573139

[B19] Hausding M. Jurk K. Daub S. Kröller-Schön S. Stein J. Schwenk M. (2013). CD40L contributes to angiotensin II-induced pro-thrombotic state, vascular inflammation, oxidative stress and endothelial dysfunction. *Basic Res. Cardiol.* 108:386. 10.1007/s00395-013-0386-5 24061433

[B20] Hu L. Zhu D. Yu Z. Wang J. Q. Sun Z. Yao T. (2002). Expression of angiotensin II type 1 (AT1) receptor in the rostral ventrolateral medulla in rats. *J. Appl. Physiol.* 92 2153–2161. 10.1152/japplphysiol.00261.2001 11960969

[B21] Jia G. Cheng G. Gangahar D. M. Agrawal D. K. (2008). Involvement of connexin 43 in angiotensin II-induced migration and proliferation of saphenous vein smooth muscle cells via the MAPK-AP-1 signaling pathway. *J. Mol. Cell. Cardiol.* 44 882–890. 10.1016/j.yjmcc.2008.03.002 18405916 PMC2765202

[B22] Lassegue B. San M. A. Griendling K. K. (2012). Biochemistry, physiology, and pathophysiology of NADPH oxidases in the cardiovascular system. *Circ. Res.* 110 1364–1390. 10.1161/circresaha.111.243972 22581922 PMC3365576

[B23] Li Y. Q. Li X. B. Guo S. J. Chu S. L. Gao P. J. Zhu D. L. (2013). Apocynin attenuates oxidative stress and cardiac fibrosis in angiotensin II-induced cardiac diastolic dysfunction in mice. *Acta Pharmacol. Sin.* 34 352–359. 10.1038/aps.2012.164 23334241 PMC4002490

[B24] Liguz-Lecznar M. Skangiel-Kramska J. (2007). Vesicular glutamate transporters (VGLUTs): the three musketeers of glutamatergic system. *Acta Neurobiol. Exp.* 67 207–218. 17957901 10.55782/ane-2007-1649

[B25] Liu D. Gao L. Roy S. K. Cornish K. G. Zucker I. H. (2006). Neuronal angiotensin II type 1 receptor upregulation in heart failure: activation of activator protein 1 and jun N-terminal kinase. *Circ. Res.* 99 1004–1011. 10.1161/01.res.0000247066.19878.93 17008603

[B26] Liu D. Gao L. Roy S. K. Cornish K. G. Zucker I. H. (2008). Role of oxidant stress on AT1 receptor expression in neurons of rabbits with heart failure and in cultured neurons. *Circ. Res.* 103 186–193. 10.1161/circresaha.108.179408 18566341 PMC2574821

[B27] Mills K. T. Bundy J. D. Kelly T. N. Reed J. E. Kearney P. M. Reynolds K. (2016). Global disparities of hypertension prevalence and control: a systematic analysis of population-based studies from 90 countries. *Circulation* 134 441–450. 10.1161/circulationaha.115.018912 27502908 PMC4979614

[B28] Minas J. N. Thorwald M. A. Conte D. Vázquez-Medina J. Nishiyama A. Ortiz R. M. (2015). Angiotensin and mineralocorticoid receptor antagonism attenuates cardiac oxidative stress in angiotensin II-infused rats. *Clin. Exp. Pharmacol. Physiol.* 42 1178–1188. 10.1111/1440-1681.12473 26234762 PMC4596772

[B29] Omura T. Yoshiyama M. Matsumoto R. Kusuyama T. Enomoto S. Nishiya D. (2005). Role of c-jun NH2-terminal kinase in G-protein-coupled receptor agonist-induced cardiac plasminogen activator inhibitor-1 expression. *J. Mol. Cell. Cardiol.* 38 583–592. 10.1016/j.yjmcc.2005.01.008 15808835

[B30] Oshima N. Kumagai H. Onimaru H. Kawai A. Pilowsky P. M. Iigaya K. (2008). Monosynaptic excitatory connection from the rostral ventrolateral medulla to sympathetic preganglionic neurons revealed by simultaneous recordings. *Hypertens. Res.* 31 1445–1454. 10.1291/hypres.31.1445 18957816

[B31] Pan Y. X. Gao L. Wang W. Z. Zheng H. Liu D. Patel K. P. (2007). Exercise training prevents arterial baroreflex dysfunction in rats treated with central angiotensin II. *Hypertension* 49 519–527. 10.1161/01.hyp.0000256955.74461.93 17224469 PMC1904508

[B32] Peng J. Wang Y. K. Wang L. G. Yuan W. J. Su D. F. Ni X. (2009). Sympathoinhibitory mechanism of moxonidine: role of the inducible nitric oxide synthase in the rostral ventrolateral medulla. *Cardiovasc. Res.* 84 283–291. 10.1093/cvr/cvp202 19535378

[B33] Reja V. Goodchild A. K. Phillips J. K. Pilowsky P. M. (2006). Upregulation of angiotensin AT1 receptor and intracellular kinase gene expression in hypertensive rats. *Clin. Exp. Pharmacol. Physiol.* 33 690–695. 10.1111/j.1440-1681.2006.04420.x 16895541

[B34] Rincón J. Correia D. Arcaya J. L. Finol E. Fernández A. Pérez M. (2015). Role of angiotensin II type 1 receptor on renal NAD(P)H oxidase, oxidative stress and inflammation in nitric oxide inhibition induced-hypertension. *Life Sci.* 124 81–90. 10.1016/j.lfs.2015.01.005 25623850 PMC6037991

[B35] Ross C. A. Ruggiero D. A. Park D. H. Joh T. H. Sved A. F. Fernandez-Pardal J. (1984). Tonic vasomotor control by the rostral ventrolateral medulla: effect of electrical or chemical stimulation of the area containing C1 adrenaline neurons on arterial pressure, heart rate, and plasma catecholamines and vasopressin. *J. Neurosci.* 4 474–494. 10.1523/JNEUROSCI.04-02-00474.1984 6699683 PMC6564896

[B36] Ushio-Fukai M. Alexander R. W. (2004). Reactive oxygen species as mediators of angiogenesis signaling: role of NAD (P)H oxidase. *Mol. Cell. Biochem.* 264 85–97. 10.1023/B:MCBI.0000044378.09409.b5 15544038

[B37] Vigneault E. Poirel O. Riad M. Prud’Homme J. Dumas S. Turecki G. (2015). Distribution of vesicular glutamate transporters in the human brain. *Front. Neuroanat.* 9:23 10.3389/fnana.2015.00023 PMC4350397 25798091

[B38] Wakui H. Dejima T. Tamura K. Uneda K. Azuma K. Maeda A. (2013). Activation of angiotensin II type 1 receptor-associated protein exerts an inhibitory effect on vascular hypertrophy and oxidative stress in angiotensin II-mediated hypertension. *Cardiovasc. Res.* 100 511–519. 10.1093/cvr/cvt225 24189624

[B39] Wang G. Coleman C. G. Chan J. Faraco G. Marques-Lopes J. Milner T. A. (2013). Angiotensin II slow-pressor hypertension enhances NMDA currents and NOX2-dependent superoxide production in hypothalamic paraventricular neurons. *Am. J. Physiol. Regul. Integr. Comp. Physiol.* 304 R1096–R1106. 10.1152/ajpregu.00367.2012 23576605 PMC3680791

[B40] Wang H. D. Johns D. G. Xu S. Cohen R. A. (2002). Role of superoxide anion in regulating pressor and vascular hypertrophic response to angiotensin II. *Am. J. Physiol. Heart Circ. Physiol.* 282 H1697–H1702. 10.1152/ajpheart.00914.2001 11959633

[B41] Wang M. Chen D. Chen L. Cao G. Zhao H. Liu D. (2018). Novel inhibitors of the cellular renin-angiotensin system components, poricoic acids, target Smad3 phosphorylation and Wnt/β-catenin pathway against renal fibrosis. *Br. J. Pharmacol.* 175 2689–2708. 10.1111/bph.14333 29679507 PMC6003649

[B42] Wei S. G. Yu Y. Zhang Z. H. Weiss R. M. Felder R. B. (2008). Mitogen-activated protein kinases mediate upregulation of hypothalamic angiotensin II type 1 receptors in heart failure rats. *Hypertension* 52 679–686. 10.1161/hypertensionaha.108.113639 18768402 PMC2790407

[B43] Wu L. He Y. Jiang B. Sun D. Wang J. Liu M. (2015). Trends in prevalence, a wareness, treatment and control of hypertension during 2001-2010 in an urban elderly population of China. *PLoS One* 10:e0132814. 10.1371/journal.pone.0132814 26241049 PMC4524712

[B44] Xia C. Shao C. Xin L. Wang Y. Ding C. Wang J. (2008). Effects of melatonin on blood pressure in stress-induced hypertension in rats. *Clin. Exp. Pharmacol. Physiol.* 35 1258–1264. 10.1111/j.1440-1681.2008.05000.x 18637016

[B45] Xiang H. B. Liu C. Liu T. T. Xiong J. (2014). Central circuits regulating the sympathetic outflow to lumbar muscles in spinally transected mice by retrograde transsynaptic transport. *Int. J. Clin. Exp. Pathol.* 7 2987–2997. 25031717 PMC4097212

[B46] Xiao F. Jiang M. Du D. Xia C. Wang J. Cao Y. (2013). Orexin A regulates cardiovascular responses in stress-induced hypertensive rats. *Neuropharmacology* 67 16–24. 10.1016/j.neuropharm.2012.10.021 23147417

[B47] Zha Y. P. Wang Y. K. Deng Y. Zhang R. W. Tan X. Yuan W. J. (2013). Exercise training lowers the enhanced tonically active glutamatergic input to the rostral ventrolateral medulla in hypertensive rats. *CNS Neurosci. Ther.* 19 244–251. 10.1111/cns.12065 23521912 PMC6493496

[B48] Zhang C. Xia C. Jiang M. Zhu M. Zhu J. Du D. (2013). Repeated electroacupuncture attenuating of apelin expression and function in the rostral ventrolateral medulla in stress-induced hypertensive rats. *Brain Res. Bull.* 97 53–62. 10.1016/j.brainresbull.2013.05.013 23751198

[B49] Zhang G. X. Lu X. M. Kimura S. Nishiyama A. (2007). Role of mitochondria in angiotensin II-induced reactive oxygen species and mitogen-activated protein kinase activation. *Cardiovasc. Res.* 76 204–212. 10.1016/j.cardiores.2007.07.014 17698051

